# Facility managers’ experiences of mentorship in a district of Gauteng province, South Africa

**DOI:** 10.4102/hsag.v29i0.2598

**Published:** 2024-07-05

**Authors:** Itumeleng G. Msiza, Thanyani G. Lumadi

**Affiliations:** 1 Department of Health Studies, Faculty of Human Sciences, University of South Africa, Pretoria, South Africa

**Keywords:** experience, facility managers, mentorship, mentoring, nurse managers, primary health care

## Abstract

**Background:**

Mentoring is recommended as a method to assist nurses in developing their leadership roles. Support and mentorship of nursing managers can yield positive results for their organisations because of the leadership quality. The lack of mentorship programmes for newly appointed facility managers has an impact on the management of the facilities.

**Aim:**

This study aims to explore and describe how facility managers experience mentorship at primary health care (PHC) facilities.

**Setting:**

Participants were drawn from 11 different PHC facilities falling under the three sub-districts: Emfuleni, mid-Vaal and Lesedi.

**Methods:**

A qualitative exploratory and descriptive research design was employed to achieve the study objectives. A non-probability purposive sampling method was used to select the facility managers from three sub-districts; a signed informed consent to participate in the study was obtained from each participant. A semi-structured interview guide was used to interview purposively selected facility managers. The interviews were audio recorded and subsequently transcribed verbatim. Data were analysed using the content analysis method. Rigour was ensured, and ethical principles measures were applied.

**Results:**

Four themes emerged from the results: the experiences on mentorship, views on mentorship, barriers to mentorship and mentorship improvement strategies.

**Conclusion:**

The study found that there was a lack of formal mentoring in the district, and there is a need for benchmarking and the development of a formal mentorship programme.

**Contribution:**

The results could be useful in identifying gaps, making recommendations to nursing management and future research. They could further broaden insight into the mentoring needs of facility managers.

## Introduction

Mentorship is useful in public services as it contributes to capacity building and skills development. According to the South African Department of Public Service and Administration (DPSA) (2006:9), mentors play two broad roles, namely psychological and facilitating roles. In the psychological role, the mentor provides the mentee with emotional support. While the facilitating role focusses on transferring skills in the workplace, including training and orientating the mentees concerning the realities of their workplace to ensure maximum performance (DPSA 2006:9). Mentoring relationships are beneficial because they can lead to improved job satisfaction and performance (Mcilongo & Strydom 2021:2). Mentorship also has a significant influence on the building of leadership skills. Effective mentoring leads to the development of strong relationships (Bodilenyane & Mooketsane 2019:690). Clarifying the leadership roles of nursing managers thus contributes to their support and mentorship and can yield positive results for their organisations because of the quality of leadership (Nene 2022:2). Mcilongo and Strydom (2021:7) supported that mentoring in leadership development affects career advancement of employees positively. Bodilenyane and Mooketsane (2019:690) argued that mentoring cannot be separated from leadership because leaders in organisations have an impact on the success of their organisations. Both management and leadership are important in the delivery of effective health services. However, health facility managers in South Africa experience various challenges such as staff shortages (Mutshatshi & Munyai 2022:4). Managers often must act as substitutes in such cases resulting in them neglecting their managerial duties. That may impact the development of their management skills and knowledge negatively. Expanding management competencies can be in the form of mentoring when individuals who are experts or more knowledgeable in their fields transfer their knowledge to less knowledgeable individuals. This is of particular importance in the primary health care (PHC) setting, which was the focus of this research. According to Mutale et al. (2017:1), managers are often ill-prepared to manage complex health systems; hence mentoring is recommended as a method to assist nurses in developing their leadership roles (Dirks 2021:11).

There is a need for further research on mentoring, over and above interventions to explore the nature of mentor–mentee relationships and their influence on supporting the uptake of research evidence (Abdullah et al. 2014:298). The researcher noted the mentorship challenges experienced by facility managers in the Sedibeng District while working there as a PHC nurse. A lack of mentorship programmes for newly appointed or acting facility managers was identified as having a negative impact on the running of health care facilities in the district. Based on that observation, the researcher sought to answer the following question: What are the experiences of facility managers concerning mentorship at PHC facilities in the district of Gauteng province? The objective of the study is to explore the experiences of facility managers on mentorship at PHC facilities.

## Research method and design

### Study design

The interpretivist paradigm was applied in this research study to understand the experiences of facility managers on mentoring. A qualitative, descriptive and exploratory research design was used to respond to the study phenomenon. An exploratory research design is useful in clarifying how a phenomenon manifests itself (Hunter, McCallum & Howes 2019). This research design allowed the researcher to uncover what is not known about the topic by involving the study participants to contribute new knowledge. A qualitative descriptive design was furthermore followed as it offered an opportunity to gather rich descriptions about a little-known phenomenon. The focus on producing a rich description of the phenomenon with the aid of knowledgeable individuals offered a unique opportunity to gain inside or emic knowledge and learn how they see their world (Bradshaw, Atkinson & Doody 2017:3). The study was descriptive in nature because of its aim of focussing on experiences of mentorship; therefore, the findings of the study are used to describe the existing situation of facility managers in the district.

### Setting

The study was conducted in the district, situated in the south of Gauteng. It comprises three health sub-districts such as Emfuleni, Lesedi and Midvaal.

There are 31 clinics, four community health centres, two district hospitals and one regional hospital. The data were collected in the 11 PHC facilities.

### Study population and sampling

The target population comprised all the facility managers and acting facility managers working at PHC facilities in the district, with the accessible population being available facility managers willing to participate in the study. An estimated 35 facility managers were targeted, of which only approximately 20 were accessible. Those who were not accessible were either on leave, unavailable or not interested in participating in the study. Out of the 20 accessible facility managers, some withdrew from the study, while others lacked interest, did not honour appointment dates or were excluded based on the sample exclusion criteria that the researcher employed. The criteria for sampling participants included those employed as PHC facility managers, acting facility managers and professional nurses who were selected to act as managers for more than 3 months. Exclusion criteria were other categories of managers such as area managers, deputy directors and directors as well as PHC facility managers outside Sedibeng District.

The researcher selected participants who could provide the information required to achieve the study objectives, by using a purposive sampling technique. This method allowed the researcher to select participants who were known to be facility managers or those who have acted as facility managers in the Sedibeng District. Four clinics in each of the three sub-districts in the district were contacted to access the facility managers to obtain consent to participate in the study and to agree on interview dates, times and convenient locations. The researcher interviewed 11 facility managers from 11 different PHC facilities and data saturation was reached (Bradshaw et al. 2017:4).

### Data collection

A semi-structured interview guide was developed (DeJonckheere & Vaughn 2019:5), consisting of a prearranged set of open-ended questions. Probing was done to obtain more information from the participants during the interviews (DeJonckheere & Vaughn 2019:3). The questions in the interview guide were tested by conducting a pilot study to ensure the validity and reliability of the instrument (Buschle, Reiter & Bethmann 2021:838). Potential participants were given adequate time to decide whether they wanted to participate. They were provided with an information leaflet and advised that they could withdraw from the study at any time. Those agreeing to participate were required to sign the consent form. They also granted consent for the interviews to be recorded. Face-to-face interviews were conducted from June 2022 to February 2023. Data were collected over a period of 8 months, in the absence of coronavirus disease 2019 (COVID-19) lockdown regulations during alert level 1, adhering to all COVID-19 protocols. A digital voice recorder was used to record interviews and other non-verbal messages conveyed by the participants were handwritten in field notes. The participants were interviewed individually at the clinic or venue of their choice, which included their homes on the dates set for appointments. The main aspects covered during the interviews were facility management experiences, mentorship and support in general. Each interview lasted between 30 min and 45 min. The principal researcher is a PHC nurse with experience and was able to probe for more information from participants, as there were follow-up questions emerging between the researcher and participants during the interview dialogue. The principal researcher is experienced in working at PHC facilities, under the management of different facility managers. The researchers employed social inquiry to add value to the research and also noted their values and biases related to mentorship in PHC facilities.

### Data analysis

The content analysis method was adopted to analyse the data and concepts, categories and themes were established (Kyngas, Mikkonen & Kaariainen 2020:13). To generate the interview transcriptions, the interview data were transcribed manually from the digital voice recordings in a narrative format. That assisted in sorting and interpreting the data to extract patterns and meanings and to establish themes through coding. Furthermore, the steps of coding were adopted, and themes were identified to analyse data (Dawadi 2020:64). During that process, the researcher avoided bias by setting aside what is known about mentorship. The researcher was open to the perceptions of the participants rather than attaching her own beliefs. The researcher analysed and interpreted the meaning that the facility managers ascribed to their own experiences of being mentored in the PHC facilities. The researcher accepts and acknowledges that many interpretations of reality exist and thus made use of verbatim quotes to reference the subjective interpretation of the facility managers’ experiences on mentorship. An independent coder experienced in qualitative research did co-coding. Afterwards, the researcher and the independent coder held a meeting in order to reach a consensus.

### Ethical considerations

Permission to conduct the study was obtained from the National Health Research Database (NHRD), NHRD reference number GP 202 107 004 GP, while ethical approval was obtained from the Research Ethics Committee of the College of Human Sciences at Unisa reference number 62072161_CREC_CHS_2021. Permission to conduct the research in the district was granted by the Chief Director of Sedibeng District Health Services.

Signed informed consent was obtained from the participants who had been advised that they could withdraw from the study at any time. Anonymity was ensured by allocating participants codes to identify the information provided by participants rather than by using their names. Interviews were furthermore conducted individually in private rooms to ensure privacy, free from noise and disturbances at either the clinics or participant homes. Access to the interview venues was furthermore strictly minimised. Data were stored by transferring the voice recordings and handwritten field notes to a password-protected computer and only accessible to the researcher and those involved in data analysis. The independent coder signed a confidentiality agreement.

## Results

### Demographic data

Nine of the 11 participants interviewed were females, and two were males. Their ages ranged from 31 years to 59 years, with an average age of 44 years. The educational background of the participants varied from a 3rd year to a 4th year nursing diploma, which allowed them to register as professional nurses, to degrees in nursing management and postgraduate or post-basic diplomas in a PHC speciality. Out of the 11 participants, six had a nursing management qualification obtained either as a degree or as a diploma, while 10 participants had a PHC speciality qualification. The total nursing experience of the participants ranged from 7 years to 36 years, with an average of 20 years.

Seven participants had been appointed as facility managers, while four were acting facility managers. The experiences of those appointed or acting as facility managers during the time of the interviews ranged from 5 months to 8 months. On average, the participants had less than 5 years of experience in facility management.

### Themes and subthemes

The responses of participants were arranged into the following themes: experiences on mentorship, views on mentorship, barriers to mentorship and improvement strategies as indicated in Table 1.

**TABLE 1:** Themes and sub-themes.

Theme	Sub-themes
1. The diverse experiences of facility managers	1.1. Lack of support from area managers 1.2. The dynamic and challenging role of facility managers 1.3. Positive experiences
2. Facility manager’s views on mentorship	2.1. Positive experiences enhancing mentorship support 2.2. Improving competence 2.3. Provision of quality services
3. Barriers to mentorship	3.1. Lack of policies and guidelines 3.2. Resource constraints 3.3. Poor relationship between mentors and mentees
4. Mentorship improvement strategies	4.1. Orientation programme 4.2. Group mentorship 4.3. Formal mentorship programmes and committees 4.4. Benchmarking for best practices from other districts

### Theme 1: The diverse experiences of facility managers

In discussing their experiences as managers at PHC facilities, participants highlighted the lack of area managers’ support, the dynamic and challenging role of facility managers as well as their positive experiences as managers.

#### Sub-theme 1.1: Lack of support from area managers

Most participants mentioned that they lacked sufficient facility management knowledge because of a lack of support from area managers; this was noted as a negative experience. One of the participants had this to say:

‘It’s very difficult. There is no support from the area managers because you are only placed there to act, whereas you have no knowledge at all of what is happening around. You don’t even know which channels to follow if there is a problem.’ (Participant 3, Female, 42 years old)

Another participant who was acting as a facility manager mentioned the following:

‘The post was vacant, so I didn’t get any mentorship. The area manager only came to the clinic and asked me to act. That was it, so I had to see about most of the things, if not everything to completion.’ (Participant 9, Female, 32 years old)

Facility managers highlighted the total absence of a mentor as a negative experience, as mentioned below:

‘I didn’t have a mentor. It was a self-appointed mentor by myself, to say that I want to be like that person, I am going to follow in their footsteps, and consult with them whenever I am having challenges; but it was never someone that was appointed by the district to assist me.’ (Participant 10, Female, 31 years old)

‘Actually, I can’t talk about mentoring because there is none.’ (Participant 3, Female, 42 years old)

Lack of support and absence of mentors were also regarded as negative experiences. Participants indicated the following:

‘As a facility manager we work under the management of an area manager, there is no support at all. The area managers are there, they come, and you know, speak to us about our data, everything like that but they never contribute towards making the workspace a pleasant place to work, because if you don’t know anything and you have to find things yourself, it’s not pleasant you know. Imagine now, you are from another district, and you are here, you don’t know of anyone, who are you going to lean on, so I feel like there is a lot of lack between the relationship of a facility manager and the area managers that are put there to support us.’ (Participant 10, Female, 31 years old)

‘There is no support at all because if you are placed in the office, you go there without any knowledge.’ (Participant 3, Female, 42 years old)

A few participants identified their supervisors or area managers and their colleagues as their mentors, reporting that they received support and guidance from them. As mentioned below:

‘Yes. The area manager was my mentor. My supervisor was my mentor because she used to guide me on how things are done until I acquired the experience, then I was able to do the work alone. If I was alone, I wouldn’t know how to do it, so it would have been difficult. The mentoring meant a lot for me. Besides, my seniors and colleagues also have some experience, so they guided me on certain things that I didn’t know.’ (Participant 2, Female, 54 years old)

‘With me, when I was working, she was constantly there. She would tell me in the morning to come and plan for the day, and tell me how to run the day, how things are done, and that if I have any challenge she is there to assist. For example, if you need something from Stores, you need to call them, take charge, and tell them the challenges and what you expect.’ (Participant 7, Female, 34 years old)

Participants’ experience of limited support from the area managers was seen as a contributing factor to the challenges of mentorship. However, those who received support from their managers were grateful and considered support and guidance as mentorship.

#### Sub-theme 1.2: The dynamic and challenging role of facility managers

Participants found the experience of transitioning roles challenging, specifically when moving from clinical duties to managerial duties. This challenge was attributed to a lack of consistency and preparation.

According to one participant, the role of a facility manager is:

‘Challenging, it keeps on changing from time to time’ (Participant 2, Female, 54 years old)

Most facility managers were of the view that lack of preparation or special training was a further challenge, as mentioned below:

‘It’s challenging; a new challenge every day, and you lack on the job. I can safely say you are never prepared to be a facility manager. There is no preparation, or special training. You just have to move from being a professional nurse or a clinician, to being a facility manager.’ (Participant 1, Male, 33 years old)

‘It was draining, I didn’t have anyone guiding me, I didn’t have a strategy on what to do. Now, that’s when I start reflecting that if I had somebody to mentor me, then I would have had maybe my team to direct on what to do and work together. It was quite difficult, I was just working for the sake of working.’ (Participant 9, Female, 32 years old)

Another participant had this to say about the challenges experienced:

‘It is not easy because when you are a nurse practitioner, most of the things you are introduced to are patient-based. When you come this side, there is a lot of admin, and you have to transition just like that. It’s not easy because even when the patients and colleagues see you, they don’t see a manager. They still call you to do some things but when it comes to management things, they step away. You are the one that has to deal with it.’ (Participant 11, Female, 35 years old)

#### Sub-theme 1.3: Positive experiences

Facility managers noted independent learning as positive and some acknowledged the guidance received from their colleagues as beneficial mentorship. Explaining her positive perception of independent learning, one participant noted the following about her supervisor:

‘She’s that kind of a person that says if you need something, you must do it first. If you fail, you come to me, that disturbed me at first. I felt like she doesn’t want to help or mentor me, but as time went on, I realised that it is not such a bad thing because I realised that if you do something yourself, you learn better than if it is given to you on a silver platter.’ (Participant 11, Female, 35 years old)

Participants believed that mentoring someone by giving them proper advice and support needed to be effective in their position helps them to make good decisions:

‘I believe that when someone is given proper advice and the support that they need, they are going to be effective and be able to make good decision in terms of management and leadership. They will also be confident in doing what they need to do; hence, there won’t be unnecessary and costly mistakes because they will be able to direct the staff accordingly and reach their vision and missions as set by the organisational structures.’ (Participant 8, Female, 55 years old)

Another participant added that mentoring provides career growth for individuals:

‘When you mentor the sisters, you mentor to see them as the next managers. So, when they do things, don’t just do them for the sake of just doing. You mentor people to see the benefits of what they are doing, and to see the difference. When I arrived at our facility, I instilled the culture of mentorship because mentoring is far different from managing. There is a difference between mentoring and managing.’ (Participant 5, Female, 49 years old)

Other participants asserted that mentoring empowers individuals and empowers them to become innovative:

‘Once you are mentored or during the mentorship, you become innovative and empowered. When you wake up in the morning with a goal to achieve based on what the mentor taught you to do. For example, how to make medicine orders. You become empowered, even to also want to be like my mentor.’ (Participant 9, Female, 32 years old)

### Theme 2: Facility manager’s views on mentorship

The participants were asked to give their general understanding of the term mentorship and to highlight whether they were mentored in their positions as facility managers. Mentorship was associated with support, making others better people and improving the quality of health care.

#### Sub-theme 2.1: Positive experiences enhancing mentorship support

Facility managers were of the view that support is an important aspect of mentorship. The following statements regarding support were indicated:

‘Mentorship means that I got someone who supports me and walks me through to the work that I do daily, monthly and yearly. So, people who will support me and show me the way. That is mentoring. They guide me with their experience.’ (Participant 2, Female, 54 years old)

‘Mentorship to me says guidance, says this is what I have been doing and this is what you should do be doing, this is what is expected from you, and I can assist you up to this much. I can teach you 1, 2, 3, 4. I can teach you what to expect and how to deal with some staff, of course you won’t be fed with a silver spoon, this is a professional platform, I have been here before so you can expect this and that and if you struggle with this I can come and do the walk-through steps with you and then leave you on the way to be on your own.’ (Participant 1, Male, 33 years old).

The aforementioned verbatim indicates the significance of having a mentor who will guide you to become an independent professional.

#### Sub-theme 2.2: Improving competence

Participants viewed mentorship as an important aspect in improving the competence of facility managers; one participant mentioned the following statement:

‘Facility managers I feel like they should be equipped on mentoring more than anything else, like managing whatever they can but mentoring is the most important thing because when the people come to you, they are orientated and inducted, so your role is to mentor them into being the better people or in better rendering the services. Because somebody who is not mentored, even the services might not be eh up to scratch because we looking at the quantity neh, the quality, not the quantity of the services. We want the quality, we can see 17 people neh but if out of 17 people, 10 people complained about us, it does not make any sense for me, so the mentoring is very important, I feel like in the district, the facility managers are just employed and then left there on their own and not done anything.’ (Participant 5, Female, 49 years old)

#### Sub-theme 2.3: Provision of quality services

Another participant was of the view that mentorship can provide facility managers in the health department with better management skills as discussed here:

‘The benefits of being … for mentoring is that we save time and then you easily reach your goals and also the organizational goals and visions are easily met, so we can happen to provide a good and a quality service because now you know what you are doing, you are equipped and also you understand clearly as a human being who led people and there’s harmony that’s going to produce the quality that we are expecting in every situation that we are dealing with in everyday life and also with us we know that health department is facing a lot of scrutiny outside because people don’t know what to do.’ (Participant 8, Female, 55 years old)

Mentorship has many benefits and hence the importance of ensuring that the programme is developed and implemented in health care facilities.

### Theme 3: Barriers to mentorship

Participants highlighted the lack of policies and formal mentoring programmes in the district, lack of resources including financial and human resources and poor relationships as barriers to mentorship.

#### Sub-theme 3.1: Lack of policies and guidelines

One participant said that there were no policies or guidelines to support mentoring in the district:

‘There is no policy that I know of on any mentoring programme for the newly appointed facility managers at the district. I think they are not available because it is not practiced according to my experience.’ (Participant 8, Female, 55 years old)

Another participant added the following regarding the availability of policies and guidelines in the district:

‘There is no SOP’s or guidelines that I know, that talks to mentoring at all. I do not know maybe it’s there, it exists but I have never heard of it, yaah ….’ (Participant 1, Male, 33 years old).

While another participant just shook her head in response to having policies and guidelines in the district and replied:

‘mmmm … ’ (Participant 11, Female, 35 years old).

#### Sub-theme 3.2: Resource constraints

Resource constraints including financial and human resource constraints were regarded as barriers to having a formal mentorship programme as indicated below:

‘I think the barriers are, I’ll have to take it to the budget because if you have to have a mentor, you can’t have a mentor who is actively working somewhere else, I think you must have eh program that is strictly mentorship and if we have to do that it means we have to hire people for that and already for the existing eh professionals we are not even getting paid enough you know, so, I don’t think the district has another budget to budget for a completely new program of mentoring, you know or mentorship.’ (Participant 10, Female, 31 years old)

#### Sub-theme 3.3: Poor relationship between mentors and mentees

It was also argued that an unsound relationship between a mentor and a mentee because of personality differences could be a challenge to the mentoring process:

‘Well, the basic one that I will think of is the clash of personalities because when it comes to mentoring, there is also criticism that comes along. So, if a personality cannot take criticism, it sort of hinders the progress because you are trying to get the information to the next person, but they are not taking it.’ (Participant 7, Female, 34 years old)

Another participant was of the view that area managers’ lack of support contributes to their poor relationships:

‘As a facility manager we work under the management of an area manager, there is no support at all, the area managers are there, they come, and you know, speak to us about our data, everything like that but they never contribute towards making the workspace a pleasant place to work because if you don’t know of anyone, who are you going to lean on, I feel like there is a lot of lack between the relationship of a facility manager and the area managers that are put there to support us.’ (Participant 10, Female, 31 years old)

### Theme 4: Mentorship improvement strategies

The participants argued that structured mentorship programmes such as orientation programmes, group mentoring, formal committees and benchmarking would improve the leadership skills of managers.

#### Sub-theme 4.1: Orientation programme

Participants emphasised the importance of having orientation programmes for facility managers within the district and the following were mentioned:

‘Mentoring should be part of the induction programme and it should be done at the teaching and staff development. It should be part of the staff development department and I will encourage the district to include this under their policy ….’ (Participant 8, Female, 55 years old)

‘I think the district should have its own programs that are mandatory to say this is … Programs that will form part of induction into management to say each and every manager before they can even start in their office, they should be program 1, program 2, program 3 that prepares you to resume a position of a manager, I think that is my recommendation, not maybe to depend on external companies of facilities from outside where you have to be sent to a certain course but there should be a program in the district, a mandatory one for everyone one.’ (Participant 1, Male, 33 years old)

#### Sub-theme 4.2: Group mentoring

One participant felt that having a group of well-experienced facility managers with those who are still new in the profession could help to improve the mentoring needs in the district:

‘If I have a problem, we work together as cluster managers. We asked the older sisters to meet with us at least once or twice a month so that they mentor us.’ (Participant 11, Female, 35 years old)

#### Sub-theme 4.3: Formal mentorship programmes or committees

Facility managers noted the importance of having a formal mentoring programme to improve mentorship in the district as mentioned by a participant below:

‘I will encourage mentoring. If we had a formal mentoring programme not only for the facility managers, but for all the categories so that the roles are clear, and the people feel guided.’ (Participant 1, Male, 33 years old)

Another participant supported the existence of a formal committee focussing on mentorship to improve mentoring in the district:

‘I think maybe there should be an official committee of some sort that deals specifically with mentoring and support. There should be the dissemination of information on the programme, and to also reassure the managers that seeking mentorship does not mean that you are lacking, but that you seek to develop and grow in the profession so that you can grow others too.’ (Participant 7, Female, 34 years old)

#### Sub-theme 4.4: Benchmarking for best practices from other districts

The importance of benchmarking from other districts and exploring other mentors’ and mentees’ experiences were mentioned as important when developing a formal mentorship programme.

Only one participant spoke about this strategy and in her statement, she mentioned the following:

‘I think eh the district can eh, can benchmark from some of the district that are doing the mentoring program or even consult people who know better and then they can ask maybe the managers what is their experiences, maybe if they can get feedback from them really what has transpired during the transition, the problems and the challenges that they have, they can develop a plan to be able to make sure that they help them in future.’ (Participant 8, Female, 55 years old)

### Measures of trustworthiness

Rigour was achieved through credibility, transferability, dependability, confirmability and authenticity, the criteria that were first addressed by Lincoln and Guba (Kyngas et al. 2020:42). Concerning credibility, the researcher established a rapport before commencing with the interviews by developing a trusting relationship, showing empathy and allowing at least two participants to verify the accuracy of the interview transcriptions. To ensure transferability, sufficient descriptive data were provided for the study to be used as a reference for other proposed studies in different contexts. Even though data were collected in different months to accommodate participants’ availability, the interviews yielded the same results, as the participants gave the same responses. That reflected dependability which was further guaranteed by having an independent coder co-coding the raw data electronically (Bradshaw et al. 2017:6). For confirmability, a voice recorder was used, ensuring that the data represented the information provided by the participants without being tainted by the researcher’s opinions or views during transcription (Kyngas et al. 2020:42). The authenticity of the study was ensured by faithfully interpreting the participants’ real-life experiences, without manipulating the findings in any way. This was achieved by using verbatim quotes (Kyngas et al. 2020:42).

## Discussion

Four themes emerged from the study, which are diverse experiences of facility managers, facility managers’ views on mentorship, barriers to mentorship and mentorship improvement strategies. A total of 11 participants formed part of the study. Just above half of the participants had management qualifications. Despite having these qualifications, they still reported challenges in their experiences of managing the facility. According to the systematic review of Kakemam et al. (2020:66), managers can develop themselves professionally in several ways including formal education at universities in the management field, in-service training and in-house use of mentors, study groups, intensive training sessions and seminars. The findings of this study correlate with those of Kakemam et al. (2020:66), as managers expressed a need to develop themselves professionally through structured and group mentoring as well as formal committees and orientation programmes. A study carried out in Kwa-Zulu Natal province found that a tertiary management qualification is not a prerequisite for being appointed to a leadership position; however, they recommend that a formal leadership qualification in management should be a requirement for unit managers role (Naicker & Hoque 2017:324).

On average, the participants in this study had fewer than 5 years of experience in management posts, which makes them ideal candidates for mentoring. A study by Kramer et al. (2021:21) found that experienced nurses had a professional responsibility to allocate their time to share knowledge and assist others to grow in the nursing profession. In this research study, participants verbalised concerns regarding the lack of support from managers.

The results of the study showed that most study participants had not received formal mentorship since their appointment as facility managers. To date, there are no formal mentorship programmes in the district to support people in leadership positions. The participants did, however, mention informal, self-created mentorship. This study’s results correlate with the findings of a study by Hubbard Murdoch et al. (2021:10) that mentorship was not specifically encouraged within the study area’s institutional culture. Participants in this study were not in favour of self-appointed mentors. However, in contrast, Ssemata et al. (2017:6) found the relationship between mentor and mentees is successful if mentees select their own mentors. In their study, Mcilongo and Strydom (2021:3) stressed the need for mentoring policies to avoid mentors only selecting the mentees that they have something in common with and including all candidates eligible for mentoring. Relevant to this, the managers targeted in this study indeed attended induction programmes. However, such programmes are aimed at everyone who gets appointed and does not necessarily focus on the specific needs of managers.

All participants in this study believed that mentorship has great benefits, such as the transfer of skills and knowledge to their subordinates, and that it influences them positively to perform better as managers. The findings of this study are supported by those of Giacumo, Chen and Seguinot-Cruz (2020:1) that formal mentoring training programmes are linked to the performance improvement of individuals in the workplace. The findings also correlate with a study that described mentoring as having a significant connection with improved skills pertinent to academic roles (Hubbard Murdoch et al. 2021:2), which further links up with the findings of this study where the participants mentioned that mentoring supports personal growth and career development.

Participants highlighted the improvement in quality health care as one of the benefits of mentoring in health care facilities, which correlates with the results of a study by Goyet et al. (2020:8) that mentoring of nurses with facility mentoring improves clinical competence in maternity care. In this study, one participant identified the clash of personalities as one of the factors that can hinder successful mentorship. A total of 57.9% of participants in a Nigerian study reported personality clashes as a barrier to mentorship (Ughasoro et al. 2022:218). Another study by Morgan et al. (2018:4) found strong mentor–mentee relationships based on mutual respect and trust to be valuable. Most participants in this study did not mention their area managers as their role models contrary to the study by Ssemata et al. (2017:4), which reported that mentors are role models providing guidance and support.

Mcilongo and Strydom (2021:8) stated that ‘mentorship training could be done by designing an induction plan for managers, which would ensure that managers are well equipped to assist less-experienced managers’. In this regard, the study participants believed that their confidence as facility managers would be boosted by improving mentorship. Yet only one participant reported participation in a mentorship programme arranged by the district. She confirmed that the course indeed prepared her to be a leader and a manager. They were, for example, taught how to handle difficult situations, which helped in shaping her leadership skills.

A study by Nagle, Omonaiye and Bennet (2021:5) found that participants indicated that administrative responsibilities were taking more time from clinical time. Participants furthermore believed that the lack of support from their area managers worsened the situation. These results are supported by a study conducted in the Northwest province of South Africa, which found a lack of supportive supervision of facility managers by local area managers (Serapelwane & Manyedi 2020:14). According to Mutshatshi and Munyai (2022:2), inadequate human resources in rural areas hinder the integration and implementation of comprehensive management at PHC clinics. The findings of this study could not be exempted from these shortages, as the participants mentioned resource constraints such as human resources and financial resources as barriers to effective mentorship programmes in the Sedibeng district. The district, which comprises Emfuleni sub-district is mainly urban in nature, and the Lesedi and Midvaal sub-districts are largely rural (Sedibeng District Spatial Development Framework 2014:44). Furthermore, Morgan et al. (2018:4) found staff shortages as a barrier to care provision and mentorship.

Participants recommended structured mentorship programmes, group mentoring, orientation programmes and formal committees as improvement strategies for a mentorship programme that correlates with the findings of a study by Hookmani et al. (2021:11) where improvement strategies such as formal and informal in-service training, mentoring of head nurse by senior nursing team and orientation of mentees were also recommended. Furthermore, one participant in this research study recommended benchmarking to develop a formal mentorship programme. Mremi et al. (2023:5) also found that being informed about previous experiences including successes and challenges can improve the mentorship programme.

### Strengths and limitations

The methodology of the study allowed the participants to express their experiences and views on mentoring within their work context. Most facility managers were reluctant to participate in the study despite talking about the challenges experienced in their roles anecdotally. Full participation might have added new dimensions to the findings.

### Recommendations

Mentorship should form part of nursing education and training. Development of mentorship policies, formal induction and formal mentorship programmes for the new managers and evaluation of the process were recommended. Benchmarking as well as consultation with PHC experts in mentorship should be considered. Challenges raised in this study should form part of the research to be conducted in developing formal mentoring programmes for nurse managers.

## Conclusion

The findings of the study revealed that there is a lack of formal mentoring and managerial support in the district. The positive experiences mentioned include independent learning and guidance from colleagues and career growth, whereas negative experiences include a lack of policies, lack of support, lack of financial and human resources and induction or orientation programmes. Participants highlighted the need to have formal mentorship structures and support to guide them in their managerial roles.
